# Macrophage-Specific ApoE Gene Repair Reduces Diet-Induced Hyperlipidemia and Atherosclerosis in Hypomorphic *Apoe* Mice

**DOI:** 10.1371/journal.pone.0035816

**Published:** 2012-05-14

**Authors:** Nathalie Gaudreault, Nikit Kumar, Victor R. Olivas, Delphine Eberlé, Joseph H. Rapp, Robert L. Raffai

**Affiliations:** Department of Surgery, University of California San Francisco, VA Medical Center, San Francisco, California, United States of America; Thomas Jefferson University, United States of America

## Abstract

**Background:**

Apolipoprotein (apo) E is best known for its ability to lower plasma cholesterol and protect against atherosclerosis. Although the liver is the major source of plasma apoE, extra-hepatic sources of apoE, including from macrophages, account for up to 10% of plasma apoE levels. This study examined the contribution of macrophage-derived apoE expression levels in diet-induced hyperlipidemia and atherosclerosis.

**Methodology/Principal Findings:**

Hypomorphic apoE (*Apoe* ^h/h^) mice expressing wildtype mouse apoE at ∼2–5% of physiological levels in all tissues were derived by gene targeting in embryonic stem cells. Cre-mediated gene repair of the *Apoe* ^h/h^ allele in *Apoe* ^h/h^LysM-Cre mice raised apoE expression levels by 26 fold in freshly isolated peritoneal macrophages, restoring it to 37% of levels seen in wildtype mice. Chow-fed *Apoe* ^h/h^LysM-Cre and *Apoe* ^h/h^ mice displayed similar plasma apoE and cholesterol levels (55.53±2.90 mg/dl versus 62.70±2.77 mg/dl, n = 12). When fed a high-cholesterol diet (HCD) for 16 weeks, *Apoe* ^h/h^LysM-Cre mice displayed a 3-fold increase in plasma apoE and a concomitant 32% decrease in plasma cholesterol when compared to *Apoe* ^h/h^ mice (602.20±22.30 mg/dl versus 888.80±24.99 mg/dl, n = 7). On HCD, *Apoe* ^h/h^LysM-Cre mice showed increased apoE immunoreactivity in lesional macrophages and liver-associated Kupffer cells but not hepatocytes. In addition, *Apoe* ^h/h^LysM-Cre mice developed 35% less atherosclerotic lesions in the aortic root than *Apoe* ^h/h^ mice (167×10^3^±16×10^3^ µm^2^ versus 259×10^3^±56×10^3^ µm^2^, n = 7). This difference in atherosclerosis lesions size was proportional to the observed reduction in plasma cholesterol.

**Conclusions/Significance:**

Macrophage-derived apoE raises plasma apoE levels in response to diet-induced hyperlipidemia and by such reduces atherosclerosis proportionally to the extent to which it lowers plasma cholesterol levels.

## Introduction

Apolipoprotein (apo) E is a multifunctional plasma glycoprotein best known for its ability to lower plasma cholesterol and protect against atherosclerosis [1]. As a high affinity ligand for the low-density lipoprotein receptor (LDLR), the LDLR related protein (LRP) and heparan sulfate proteoglycans (HSP) apoE participates in the receptor-mediated clearance of remnant lipoproteins in the liver [1], [2]. Although the liver is the major source of plasma apoE, studies have shown that extra-hepatic sources of apoE, including from macrophages, account for up to 10% of plasma apoE levels [3], [4], [5]. Adoptive transfer studies have shown that in *Apoe* ^−/−^ mice, even a 10% restoration of plasma apoE levels by macrophages is sufficient to normalize plasma cholesterol levels and prevent the formation of atherosclerosis [6], [7]. Moreover, the absence of macrophage-derived apoE is known to reduce plasma apoE levels in both wildtype (WT) [8] and *Ldlr* ^−/−^ mice [9]. Thus, macrophage-derived apoE contributes substantially to plasma apoE levels. However, despite the well established atheroprotective properties of macrophage-derived apoE [6], [7], the consequences of its deficiency on atherosclerosis remain less well understood [8], [9], [10], [11].

Early human and animal studies demonstrated that normal plasma apoE levels far exceed the minimum amount required to regulate plasma cholesterol levels [12], [13], [14]. In addition, macrophage-derived apoE was shown to accumulate within atheroma [15] while a macrophage deficiency in apoE decreased its content within atherosclerotic lesions [9], [10]. Accordingly, the concept that apoE may possess atheroprotective roles independent of its ability to lower plasma cholesterol levels emerged. To address this question, studies were designed to produce plasma apoE levels below the threshold required to lower plasma cholesterol levels by making use of macrophage-specific transgenic and viral human apoE expression [16], [17], [18] in bone marrow transplanted *Apoe* ^−/−^ mice [16], [17], [18], [19], [20]. As such, low levels of macrophage-derived apoE significantly reduced early fatty streak formation in hyperlipidemic mice [16], [19], [20]. A primary mechanism proposed to account for the anti-atherogenic properties of macrophage-derived apoE is centered on its ability to promote cholesterol efflux from macrophage foam cells [16], [17], [20], [21], [22], [23]. Although results of these studies provided important insights into the role of macrophage-derived apoE in both plasma cholesterol homeostasis and atherosclerosis, most were conducted in irradiated *Apoe* ^−/−^ mice using either transfected or transgenic inter-species expression of human apoE. In addition to the unwanted effects of irradiation [24], [25] and inter-species differences in apoE activity [18], [20], [26], these studies made use of *Apoe* ^−/−^ mice in which the complete absence of apoE could be subject to compensatory effects in metabolism and pathology [27].

Thus, this study was designed to examine the influence of macrophage-derived apoE expression levels on the susceptibility to diet-induced hyperlipidemia and atherosclerosis in mice that also expressed sub-physiological levels of apoE in all tissues. We report the development of hypomorphic apoE mice expressing ∼2–5% of WT mouse apoE in all tissues. Conditional Cre-mediated gene repair of the hypomorphic *Apoe* allele increased apoE expression to ∼40% of normal levels in macrophages and led to robust apoE expression in liver-associated Kupffer cells and lesional macrophages when mice were fed a high cholesterol diet (HCD). Results of our study demonstrate that macrophages respond to diet-induced hyperlipidemia by raising plasma apoE levels and enriching remnant lipoproteins in apoE, thereby reducing plasma cholesterol levels. We also report that macrophage-derived apoE–dependent atherosclerosis suppression is proportional to the extent of plasma cholesterol lowering.

## Results

### Generation of Hypomorphic *Apoe* ^h/h^ Mice and Conditional Expression of apoE in Macrophages

**Figure 1:**
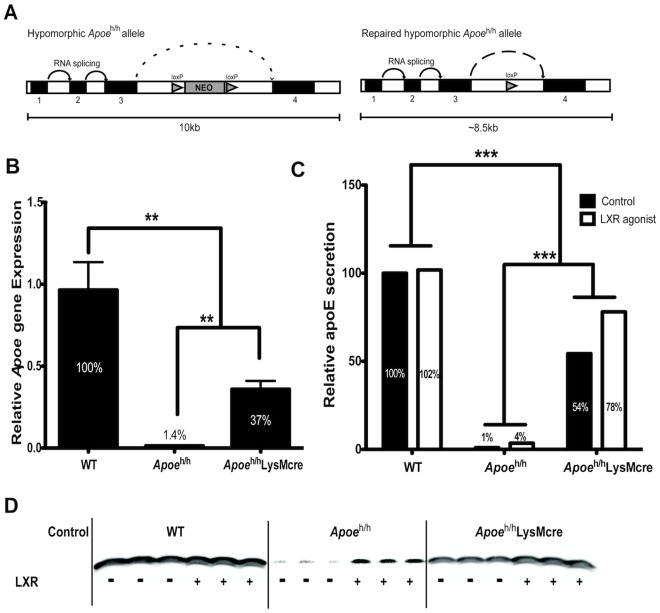
Conditional repair of the hypomorphic *Apoe* ^h/h^ allele. Hypomorphic *Apoe* gene expression and gene repair strategies (**A**). Relative *Apoe* gene expression levels in peritoneal macrophages collected from both groups of mice (n = 4; **B**). ApoE secreted by peritoneal macrophages in the presence and absence of LXR agonist pre-treatment was quantified (n = 3; **C**) from Western Blot (D), mean±sem, **p<0.01, ***p<0.001; control is media without macrophages.

The insertion of a neomycin (*neo*) resistance cassette flanked by *loxP* sites into intron 3 of the WT *Apoe* allele resulted in the attenuated expression of apoE as previously described in the apoE4-like Arg-61 *Apoe* ^h/h^ strain of mice [28]. The hypomorphic effect observed in *Apoe* ^h/h^ mice presumably arises from an aberrant mRNA splicing between *Apoe* exon 3 and the *neo* cassette (Fig. 1a). Removal of the neomycin cassette in Arg-61 *Apoe* ^h/h^ mice by Cre-mediated gene repair permanently restores normal plasma apoE levels in plasma [28]. In this study, *Apoe* ^h/h^ mice were bred to LysM-*Cre* transgenic mice that restricts the expression of *Cre* recombinase to the myeloid cell lineage, including mature macrophages [29].

We first assessed the efficiency of LysM-Cre–mediated gene repair of the hypomorphic *Apoe* allele. As shown in Figure 1B, levels of apoE mRNA in peritoneal macrophages of *Apoe* ^h/h^LysM-*Cre* mice were 26-fold higher than those of *Apoe* ^h/h^ mice; however, they were only 37% of apoE mRNA levels detected in macrophages of WT mice. This result suggests that although LysM-Cre–mediated gene repair of the hypomorphic *Apoe* allele significantly increases apoE expression levels in peritoneal macrophages, they do not return to physiological levels. We next assessed the levels of apoE secretion in supernatants of cultured peritoneal macrophages either with or without a pre-treatment with an LXR agonist (Fig. 1C&D). LXR agonist pre-treatment slightly increased the secretion of apoE in the supernatant of macrophages of *Apoe* ^h/h^ and *Apoe* ^h/h^LysM-*Cre* mice. We also determined that macrophages from each group secreted 1% and 54% respectively, of basal apoE levels detected in supernatants of WT peritoneal macrophages, mirroring the gene expression data.

### Contribution of Macrophages to Plasma apoE and Cholesterol Levels

**Figure 2:**
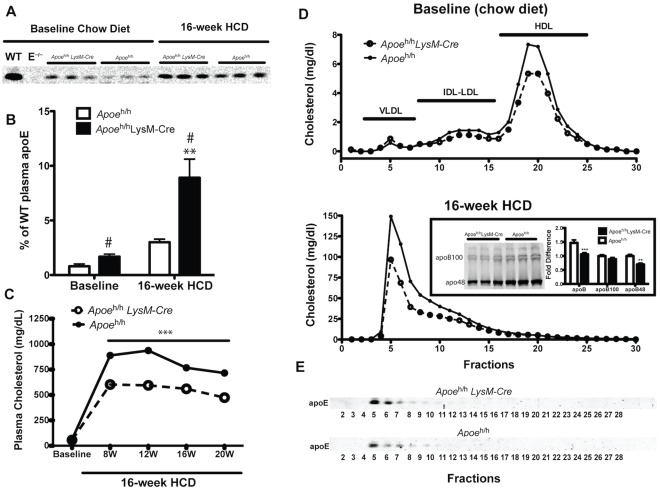
Plasma apoE and lipid levels. Representative Western blot (**A**) and quantification (**B**; n = 3) of the band intensity of plasma apoE from wild type (WT), *Apoe* ^−/−^ (E^−/−^), *Apoe* ^h/h^ and *Apoe* ^h/h^LysM-Cre mice either fed a chow diet or a high cholesterol diet (HCD); mean±sem, two-way ANOVA Bonferonni post-tests, **p<0.01 and t-test #p<0.05. Plasma cholesterol (n = 7; mean±sem, one-way ANOVA post-test, ***p<0.001; **C**) and lipoprotein cholesterol distribution (pooled of 7 mice; **D**) are shown; inset: Western blot of apoB and quantification (n = 3); mean±sem, two-way ANOVA Bonferonni post-tests, **p<0.01, ***p<0.001. Representative Western Blot of apoE distribution in lipoprotein fractions (**E**).

Because macrophage-derived apoE has been shown to reduce plasma cholesterol levels [6], [7], we investigated whether a 26-fold increase in macrophage-derived apoE expression would modulate circulating levels of plasma apoE in *Apoe* ^h/h^LysM-*Cre* mice relative to *Apoe* ^h/h^ mice. Interestingly, as shown in Figure 2A&B, *Apoe* ^h/h^LysM-*Cre* had modestly more plasma apoE than *Apoe* ^h/h^ mice when fed a chow diet, corresponding to 1.7% and 0.8% of levels seen in WT mice fed a chow diet, respectively. Following 16 weeks of HCD consumption, *Apoe* ^h/h^LysM-*Cre* mice showed a 3-fold increase in plasma apoE levels relative to *Apoe* ^h/h^ mice (Fig. 2A&B) reaching nearly 10% of WT levels.

This difference in circulating apoE levels impacted plasma cholesterol levels. In both *Apoe* ^h/h^LysM-*Cre* and *Apoe* ^h/h^ mice, plasma cholesterol levels increased from 55.53±2.90 mg/dl versus 62.70±2.77 mg/dl to 602.20±22.30 mg/dl versus 888.80±24.99 mg/dl, respectively, after 4 weeks of HCD. Despite gaining similar body weights (26.33±0.87 g versus 25.24±0.31 g at 20 weeks of age), *Apoe* ^h/h^LysM-*Cre* mice maintained 33% lower plasma cholesterol levels than *Apoe* ^h/h^ mice throughout the 16 weeks of HCD consumption (p<0.001; Fig. 2C). Plasma of both mouse models fed a chow diet displayed a normal lipoprotein cholesterol profile in which most of the plasma cholesterol was carried as high density lipoprotein (HDL; Fig. 2D, top panel). However, following 16-weeks of HCD consumption, plasma from both groups of mice displayed a lipoprotein cholesterol profile composed mainly of very low density lipoprotein (VLDL) and low density lipoprotein (LDL) with very little HDL. The increase in plasma apoE levels resulted in considerably lower levels of VLDL-cholesterol and LDL-cholesterol in *Apoe* ^h/h^LysM-*Cre* mice compared to *Apoe* ^h/h^ mice (Fig. 2D, bottom panel). Accordingly, *Apoe* ^h/h^LysM-*Cre* mice had 1.5-fold less plasma apoB, mostly as a result of a 1.4-fold reduction in plasma apoB-48 (Fig. 2D, bottom panel, inset). Western blots of fractionated plasma also revealed plasma apoE distributing mainly to the VLDL fractions (Fig. 2E). Collectively, these results suggest that the contribution of macrophage-derived apoE to the plasma apoE pool is relatively inconsequential in chow-fed mice. However, our data demonstrate that macrophages can contribute to significantly raise plasma apoE levels in mice fed a HCD. This added source of plasma apoE likely contributed to the 33% reduction in plasma cholesterol by enriching apoB-remnant lipoproteins with apoE, thereby accelerating their clearance by the liver.

### Contribution of Kupffer Cell-derived apoE to Plasma Cholesterol Homeostasis

**Figure 3:**
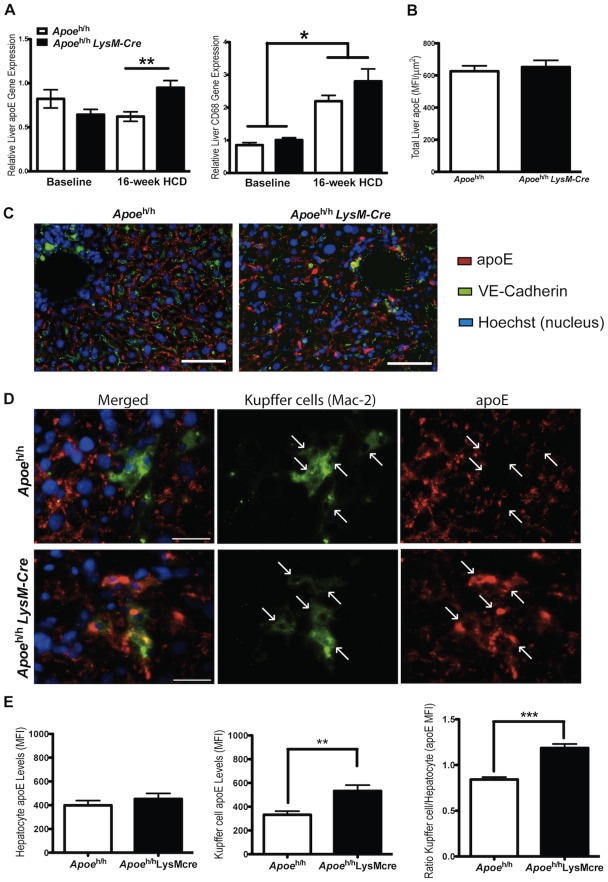
ApoE expression by liver and associated Kupffer cells. Relative CD68 (a macrophage marker) and *Apoe* gene expression were compared from whole liver extract of *Apoe* ^h/h^ and *Apoe* ^h/h^LysM-Cre mice (n = 4 for baseline and n = 7 for 16-week HCD, mean±sem, *p<0.05, **p<0.01; **A**). Relative apoE protein was also quantified and compared from immunofluorescently labeled liver cross-sections (10 µm thick) after 16 weeks of HCD (mean fluorescence intensity (MFI); n = 7, mean±sem; **B**). Representative images of whole liver cross-sections show apoE (red) surrounding hepatic sinusoidal surfaces (identified with an endothelial cell marker: VE-cadherin in green), **C**; scale bar = 100 µm). Higher resolution images demonstrate higher apoE MFI associated with Kupffer cells in *Apoe* ^h/h^LysM-Cre than in *Apoe* ^h/h^ mice (**D**; Mac-2, a macrophage marker, green; scale bar = 30 µm; white arrows point at individual kupffer cells). The apoE MFI per Kupffer cells and hepatocytes were quantified for both groups of mice and the relative ratio calculated (**p<0.01, ****p<0.0001; **E**).

We next wondered whether LysM-Cre–mediated gene repair of the *Apoe* ^h/h^ allele would increase apoE expression levels in Kupffer cells, and whether this additional source of apoE could have contributed to raise plasma apoE levels and reduce diet-induced hypercholesterolemia in *Apoe* ^h/h^LysM-*Cre* mice. To test this hypothesis, apoE mRNA expression levels and immunohistological detection of apoE were performed in liver specimens obtained from both mouse models. We detected a 1.5-fold increase in apoE mRNA levels in liver extracts of *Apoe* ^h/h^LysM-*Cre* mice compared to those of *Apoe* ^h/h^ mice when both were fed a HCD for 16-week (p<0.05; Fig. 3A, first panel). While no difference in mRNA expression levels of a macrophage-specific marker CD68 were found between the two groups, a 2.2-fold increase in CD68 was detected in the livers of mice fed a HCD compared to their baseline levels (p<0.05; Fig. 3A, right panel).

In addition to the overall increase in liver apoE mRNA levels, liver cross-sections consistently displayed regions with enhanced apoE immunoreactivity. This additional apoE did not appear to be associated with hepatic sinusoidal surfaces (identified with an endothelial cell marker) nor to be derived from hepatocytes. Quantification of apoE immunofluorescence levels from whole liver cross-sections showed relatively similar levels of apoE accumulation in liver of *Apoe* ^h/h^LysM-*Cre* and *Apoe* ^h/h^ mice (Fig. 3 B&C). We then examined at higher resolution the apoE immunofluorescence associated with Kupffer cells as identified with Mac-2, a macrophage marker (Fig. 3D). While all of the Mac-2 positive cells colocalized with a uniformly dim level of apoE staining in *Apoe* ^h/h^ mouse liver, most Mac-2 positive cells in *Apoe* ^h/h^LysM-*Cre* mouse liver colocalized with bright apoE staining. Quantification of the mean fluorescence intensity of individual cells between *Apoe* ^h/h^LysM-*Cre* and *Apoe* ^h/h^ mice confirmed similar level of apoE in hepatocytes but higher levels of apoE in Kupffer cells of *Apoe* ^h/h^LysM-*Cre* compared to *Apoe* ^h/h^ mice (Fig. 3E).

### Effect of Macrophage-derived apoE Expression Levels on Atherosclerosis

**Figure 4:**
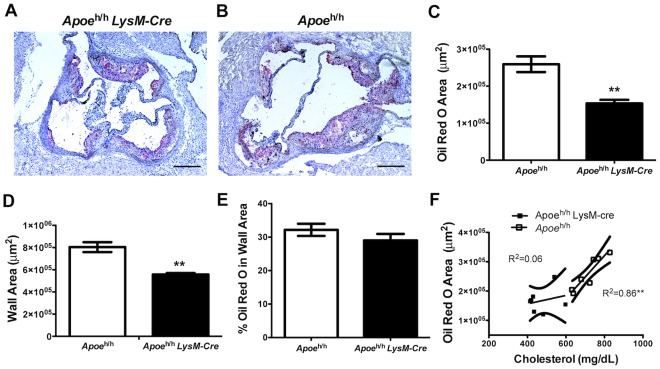
Macrophage-derived apoE levels in atherosclerosis. Histological sections of oil-red-O stained aortic roots from *Apoe* ^h/h^LysM-Cre (**A**) and *Apoe* ^h/h^ mice (**B;** scale bar = 250 µm). Quantification of oil-red-O positive area (n = 7; mean±sem, **P<0.01; **C**), aortic wall area (**D**), and % of oil-red-O (**E**). Correlation analysis between oil-red-O area and plasma cholesterol (n = 14; **P<0.01; **F**).

We next assessed the contribution of macrophage-derived apoE expression levels on atherosclerosis in *Apoe* ^h/h^ and *Apoe* ^h/h^LysM-*Cre* mice fed a HCD. As shown in Fig. 4, the aortic root oil-red O positive area was 35.5% smaller in *Apoe* ^h/h^LysM-*Cre* mice than in *Apoe* ^h/h^ mice (167×10^3^±16×10^3^ µm^2^ versus 259×10^3^±56×10^3^ µm^2^; p<0.01; Fig. 4A,B&C). However, normalizing the oil-red O positive area to that of the aortic root wall area (Fig. 4D) revealed that in both groups of mice, the atheroma within the aortic root contained the same percentage of neutral lipids (Fig. 4E). In addition, in *Apoe* ^h/h^ mice but not in *Apoe* ^h/h^LysM-*Cre* mice, the oil-red O positive area was directly proportional to plasma cholesterol levels (Fig. 4F; r^2^ = 0.86, p<0.01, and r^2^ = 0.06, respectively).

**Figure 5:**
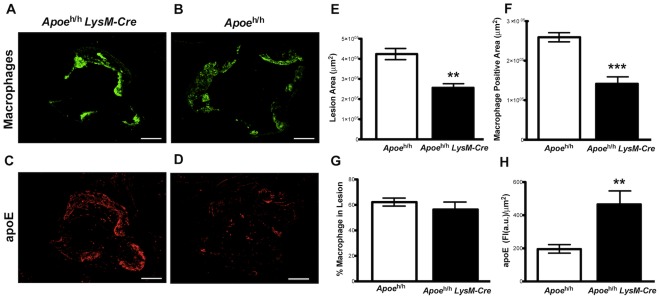
Lesional macrophage-derived apoE. Immunofluorescent images of *Apoe* ^h/h^LysM-Cre (**A,C**) and *Apoe* ^h/h^ mice (**B,D**; scale bar = 250 µm) aortic roots; anti-Mac-2 (green), and apoE (red). Quantification of lesion area (**E**), macrophage positive and % of area (**F–G**); n = 7. Quantification of apoE fluorescence intensity (FI), (**H)**; n = 7, mean±sem, *P<0.05, **P<0.01, ***P<0.001.

Additional characterizations of aortic root atheromas with immunofluorescence microscopy (Fig. 5) revealed 46% less macrophage positive area in *Apoe* ^h/h^LysM-*Cre* mice than in *Apoe* ^h/h^ mice (141×10^3^±16×10^3^ µm^2^ versus 259×10^3^±12×10^3^ µm^2^, p<0.01; Fig. 5A,B&F). However, when normalized to the aortic root intimae area (Fig. 5E), the lesions in both groups of mice contained the same percentage of macrophages (Fig. 5G). We also observed a 2.4-fold increase in apoE fluorescence intensity within the atheroma of *Apoe* ^h/h^LysM-*Cre* mice compared to that of *Apoe* ^h/h^ mice (465.3±81.2a.u./µm^2^ versus 190.8±25.9a.u./µm^2^, p = 0.01; Fig. 5C,D&H).

**Figure 6:**
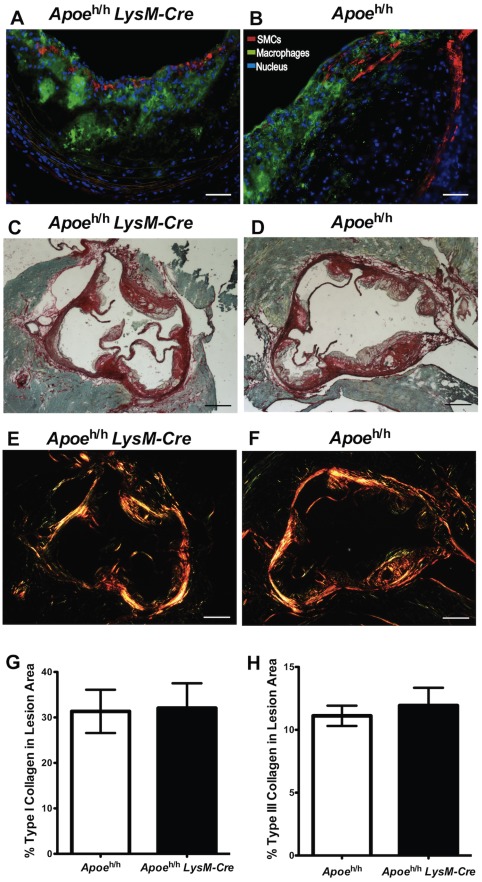
Atherosclerotic plaque composition. Immunofluorescent images of *Apoe* ^h/h^LysM*-Cre* (**A**) and *Apoe* ^h/h^ mice (**B**; scale bar = 50 µm) aortic roots; anti-smooth-muscle-cell-α-actin (SMCs, red), anti-Mac-2 (Macrophages, green) and nuclei (blue). Sirius Red stained aortic roots (brightfield (**C**–**D**) and polarized light (**E**–**F**)) from *Apoe* ^h/h^LysM-Cre (**C,E**) and *Apoe* ^h/h^ mice (**D,F**); (scale bar = 250 µm). Collagen quantification (G–H, n = 7, mean±sem).

To determine whether macrophage-derived apoE in atheroma had a local effect on plaque composition, we assessed the presence of smooth muscle cells and collagen levels in aortic root lesions of both mouse models. As shown in Figure 6A&B, the immunoreactivity for smooth muscle α-actin in the intimae of the lesions confirmed the presence of fibrous caps in both groups of mice. In addition, we found a similar percentage of total collagen content in the lesions of both *Apoe* ^h/h^LysM-*Cre* and *Apoe* ^h/h^ mice (44.0±4.6% versus 43.1±4.7%; Fig. 6C–H). Taken together, these results demonstrate that despite a 40% decrease in lesion area and two-fold increase in apoE immunoreactivity, the lesions of *Apoe* ^h/h^LysM-*Cre* mice were at a similar stage of complexity as those of *Apoe* ^h/h^ mice.

## Discussion

This study examined the contribution of macrophage-derived apoE expression levels on the modulation of diet-induced hypercholesterolemia and atherosclerosis. As such we created mouse models in which apoE was expressed either at 1.4% or 37% of normal levels in myeloid derived cells and in which all other tissues also expressed sub-physiological levels of apoE. A major finding of this study is that macrophages can respond to diet-induced hypercholesterolemia by raising plasma apoE levels and thereby reduce plasma cholesterol levels and atherosclerosis. In addition, results of this study provide evidence that apoE is produced by Kupffer cells in the setting of hypercholesterolemia. Finally, we demonstrate that a 26-fold difference in macrophage-derived apoE expression and its accumulation in atheroma are not proportionally effective in regulating the progression and complexity of advanced atherosclerosis.

Conditional Cre-mediated gene repair of the floxed hypomorphic apoE allele in *Apoe* ^h/h^ mice expressing the LysM-Cre transgene offered an opportunity to restrict an increase in apoE expression to myeloid-derived cells including peritoneal macrophages and liver-associated Kupffer cells while maintaining very low levels of apoE expression in all other tissues. LysM-Cre–mediated gene recombination has been shown to be highly efficient in mature macrophages and granulocytes and to a lower extent in CD11c+ splenic dendritic cells [29]. As shown in Fig. 1, levels of apoE mRNA in peritoneal macrophages of *Apoe* ^h/h^LysM-*Cre* mice were 26-fold higher than in *Apoe* ^h/h^ mice. However, these levels were only 37% of apoE expression levels detected in peritoneal macrophages of WT mice. This result suggests that although Cre-mediated gene repair of the hypomorphic allele significantly increases apoE expression levels in macrophages of *Apoe* ^h/h^LysM-Cre mice, they do not return to normal physiological levels. A potential explanation for this effect can be derived from studies of Xu et al. who have previously suggested that elements of retention and splicing within *Apoe* intron 3 serve to control apoE expression in neurons [30]. Thus, the additional genetic material of the *loxP* site comprised of approximately 90 base pairs that remains within *Apoe* intron 3 of the Cre-deleted hypomorphic apoE allele is likely responsible for preventing a full restoration of apoE expression levels in macrophages of *Apoe* ^h/h^LysM-*Cre* mice.

Our models offered several advantages over existing models that previously served to address the role of macrophage-derived apoE in hyperlipidemia and atherosclerosis. First and foremost, our model is the first to offer the unique possibility to study the effects of macrophage-derived apoE expression levels in the context of very low levels of hepatocyte-derived apoE expression, thus avoiding potential systemic compensations observed in conventional knockout models [27]. Comparing *Apoe* ^h/h^ and *Apoe* ^h/h^LysM-*Cre* mice allowed us to study macrophages with a 26-fold difference in apoE expression levels while all other cells expressed similarly reduced apoE expression levels. Secondly, our models avoided the use of transgenic expression of apoE from other species and the use of viral transfection systems to generate macrophages that could have varying apoE expression levels. It is noteworthy to mention that human apoE is less effective than murine apoE in mediating lipoprotein clearance in mice particularly in response to diet-induced hypercholesterolemia due to its lower affinity for murine LDL receptors [18], [20], [26]. In addition, the transfection of macrophages with an apoE-expressing retrovirus was shown to result in high degrees of variability in the level of apoE expression by macrophages [19]. In contrast, in our study, we consistently observed either 1.4% or 37% of normal apoE expression levels in peritoneal macrophages of *Apoe* ^h/h^ mice and *Apoe* ^h/h^LysM-*Cre* mice, respectively, when compared to WT mouse macrophages (Fig 1B). Moreover, all lesional macrophages appeared to uniformly express apoE in atheroma of *Apoe* ^h/h^LysM-*Cre* mice (Fig 5A&C). Thus, our model of macrophage-derived murine apoE benefits from a normal receptor-ligand affinity and a uniform expression level by all tissue macrophages including Kupffer cells and lesional macrophages.

By avoiding bone marrow transplantation as a means to restore macrophage apoE expression in *Apoe* ^−/−^ mice, our approach allowed the study of mice with no previous experimental manipulation including lethal irradiation and the expansion of marrow-derived donor cells. Thus, our models were not subject to possible effects of gamma irradiation on liver function that could cause neutral lipid accumulation; nor to possible effects on the vasculature leading to regional differences in atherosclerosis susceptibility [24], [25]. Our approach thereby enabled us to maintain an unaltered physiological environment.

Interestingly, the conditional repair of the hypomorphic allele in *Apoe* ^h/h^LysM-*Cre* mice raised plasma apoE levels only marginally (1.7% of WT) when the mice were fed a chow diet. In contrast, plasma apoE levels in *Apoe* ^h/h^LysM-*Cre* mice increased to nearly 10% of WT levels when mice were fed a HCD. This additional source of apoE did not significantly alter plasma lipid levels or the lipoprotein cholesterol profile of chow-fed mice. However, in mice fed a HCD, the more robust increase in plasma apoE contributed to reduce plasma cholesterol levels by 30% and apoB48 levels by 1.4 fold in *Apoe* ^h/h^LysM-*Cre* mice compared to *Apoe* ^h/h^ mice.

Hepatocyte-derived apoE is the main source of plasma apoE and it has been shown to be the most effective in mediating the clearance of remnant lipoproteins [31]. However, the role of Kupffer cell-derived apoE in remnant lipoprotein clearance is not well understood. It has been suggested that in the mouse, up to 20% of all tissue macrophages exist as Kupffer cells in the liver [32]. As such, it is possible that the additional amount of apoE observed in the plasma of *Apoe* ^h/h^LysM-*Cre* mice relative to *Apoe* ^h/h^ mice fed a HCD was derived at least in part from Kupffer cells. In support of this, we found a 1.5-fold increase in apoE mRNA expression in the liver of *Apoe* ^h/h^LysM-*Cre* mice relative to *Apoe* ^h/h^ mice (Fig. 3A). We reasoned that this additional amount of apoE expression could have derived from Kupffer cells. A similar expression level of the macrophage/Kupffer cell marker CD68 in the liver of both groups of mice ruled out the possibility that the increased apoE expression was simply due to a greater number of Kupffer cells present in the liver of *Apoe* ^h/h^LysM-*Cre* mice. Thus, increased expression of apoE by Kupffer cells in *Apoe* ^h/h^LysM-*Cre* mice is likely responsible for enhanced apoE expression between the two groups of mouse livers. Diet-induced hypercholesterolemia has previously been shown to induce the formation of hepatic foam cells [33]. Thus, it is tempting to speculate that Kupffer cells up-regulated apoE expression due to an excess absorption of dietary cholesterol, similar to what has been described in cholesterol-loaded macrophages [22].

Immunohistological detection of apoE in the livers of *Apoe* ^h/h^LysM-*Cre* mice confirmed that expression of Cre recombinase had remained restricted to myeloid cell lineages as hepatocytes displayed similarly reduced staining intensity for apoE in both groups of mice, while Mac-2 positive cells displayed enhanced apoE immunoreactivity only in livers of *Apoe* ^h/h^LysM-*Cre* mice. However, quantification of total anti-apoE immunofluorescence intensity in liver sections of both mouse models showed no significant differences in the content of liver apoE. It is possible that the production of apoE by hepatocytes, albeit at reduced levels and/or the low sensitivity of our immunofluorescence technique, masked the contribution of Kupffer cell-derived apoE at least at the protein level in the liver of *Apoe* ^h/h^LysM-*Cre* mice. Alternatively, it is possible that apoE produced by Kupffer cells contributed principally to enrich remnant lipoproteins with apoE that returned to the plasma thereby raising plasma apoE levels. Through further lipolytic catabolism in circulation, smaller remnant lipoproteins enriched with apoE could have been more effectively sequestered in the Space of Disse and eliminated by receptor-mediated internalization and degradation in liver hepatocytes.

Yu et al. suggested that while hepatic secretion of apoE contributes to the clearance of remnant lipoproteins, the process can occur in the absence of hepatic apoE secretion and enrichment of the space of Disse [34]. In fact, these studies found that the livers of WT and *Apoe* ^−/−^ mice had the same clearance capacity for apoE-containing remnant lipoproteins. However, livers of *Apoe* ^−/−^ mice were incapable of removing apoE-free remnant lipoproteins, while the livers of WT mice showed only 12% efficiency [34]. This suggests that apoE enrichment of remnant lipoproteins in circulation represents an important step for their clearance by the liver. The lack of a significant increase in liver-associated apoE in *Apoe* ^h/h^LysM-*Cre* mice implies that a local accumulation of apoE in their livers did not play a major role in accelerating remnant lipoprotein clearance. In contrast, our results suggest that Kupffer cell-derived apoE contributed to reduce levels of plasma apoB-48 lipoproteins by enriching them with apoE, thereby enhancing their removal from plasma in *Apoe* ^h/h^LysM-*Cre* mice fed a HCD.

Results of our study also demonstrate that atherosclerotic lesion size is directly proportional to plasma cholesterol levels in *Apoe* ^h/h^ mice but not in *Apoe* ^h/h^LysM-*Cre* mice. Similar results were uncovered when atheroma were examined for the content in smooth muscle cells and collagen, suggesting that an accumulation of apoE within lesions of *Apoe* ^h/h^LysM-*Cre* mice did not provide further protection against atherosclerosis progression than did a lower plasma cholesterol level.

While the local production of apoE by lesional macrophages has been suggested as a major source of apoE within the vessel wall [15], others have found evidence that plasma apoE diffuses into the vessel walls [26]. In *Apoe* ^h/h^LysM-*Cre* mice, the accumulation of apoE in the vessel wall likely derived from both lesional macrophages and plasma lipoprotein infiltration. The enrichment of circulating VLDL and LDL with apoE could have contributed, through their diffusion into the vascular wall, to the increased apoE content within atheroma of *Apoe* ^h/h^LysM-*Cre* mice. The presence of apoE on apoB-containing lipoproteins could have also increased their retention within the vascular wall [35], [36]. Such pro-atherogenic functions of apoE have previously been reported in mouse models of apoE-deficient macrophages [9], [11]. As such, it is conceivable that lesion-associated apoE could promote atherosclerosis by mediating the retention of lipoproteins, thereby contributing to foam cell formation in the arterial wall.

Thus, despite a 2.4-fold increase in apoE immunoreactivity in the vessel wall, we found no apparent change in the cellular composition of the atheroma between the two groups of mice. Alternatively, the use of an atherogenic diet containing cholate could have induced far too severe of a hypercholesterolemia that may have overwhelmed the anti-atherogenic properties of macrophage-derived apoE. It is also possible that the lack of an HDL component in the plasma of our hyperlipidemic mice failed to activate the reverse cholesterol transport properties of apoE. Alternatively, it is also possible that sub-physiological levels of apoE expression by macrophages are completely competent in effecting anti-atherosclerotic functions of apoE. A point of support to this is the results by Hasty el al. who demonstrated in a model of viral apoE transduction that low levels of macrophage apoE expression that did not reduce plasma cholesterol in mice nonetheless retarded atherosclerosis development. However, the effect only occurred in early lesion formation and did not persist in advanced lesions that contained other vascular cells [19]. Our data reveal that low levels of macrophage-derived apoE can effectively control the progression of advanced atherosclerosis in which cells other than foam cells accumulate.

In conclusion, results of our study demonstrate that macrophages including liver-associated Kupffer cells raise plasma apoE levels in response to diet-induced hyperlipidemia. This source of apoE enriches remnant lipoproteins with apoE and reduces plasma cholesterol levels. We also demonstrate that in our model, macrophage-derived apoE decreases atherosclerosis but only to the extent to which it lowers plasma cholesterol levels.

## Materials and Methods

### Materials

All chemicals and reagents were purchased from Sigma-Aldrich, MO, unless otherwise stated.

### Generation of Hypomorphic *Apoe* ^h/h^ Mice Expressing Wildtype Mouse ApoE

A gene targeting vector that served to create hypomorphic apoE mice expressing an apoE4-like form of mouse apoE that contains a substitution of an arginine for a threonine at residue 61 (*Apoe*R^h/h^ mice [37]), was modified to create hypomorphic apoE mice that would express reduced levels of wildtype apoE containing a threonine at position 61. Hypomorphic apoE mice expressing wildtype apoE were derived by homologous recombination of the targeting vector in embryonic stem cells as previously described [28]. Heterozygous *Apoe^h/wt^* mice were bred to C57BL/6J mice for 12 generations and offspring were intercrossed to create homozygous *Apoe^h/h^* mice.

### Conditional Cre-mediated Gene Repair of the Hypomorphic *Apoe* Allele in Macrophages

*Apoe* ^h/h^ mice were bred to LysM-*Cre* transgenic mice on a C57BL/6J background (The Jackson Laboratory, Bar Harbor, ME) that restrict the expression of *Cre* recombinase to the myeloid cell lineage, including blood monocytes, mature macrophages, and granulocytes [29]. This enables the conditional repair of the hypomorphic *Apoe* allele in macrophages. The mice were weaned at 21 days, housed in a barrier facility with a 12-h light and 12-h dark cycle, and fed a chow diet containing 4.2% fat (Harlan Teklad, Madison, WI). At 4 weeks of age, all mice were fed the “Paigen” diet containing 1.25% Cholesterol, 3.5% Coconut Oil, and 7.5% Cocao Butter (Research Diets Inc., New Brunswick, NJ) for 16 weeks. All procedures were approved by the San Francisco Veterans Administration Medical Center committee for animal care and welfare.

### Blood and Tissue Collection

Blood was collected from 4, 8, 12, 16, and 20 week-old mice fasting overnight. Mice were anesthetized with either isoflurane inhalation or Avertin (Tribromoethanol), and bled by retro-orbital or heart puncture. Plasma was isolated by centrifuging blood samples for 10 mins at 4000 rpm and stored at −80°C until performing measurements. Mice were perfused via heart puncture with ice-cold PBS containing ProtectRNA RNAse Inhibitor (Sigma; 1.5 ml/min for 10 min). The aortic root and half of the top right liver lobe were embedded in Tissue-Tek O.C.T. cryosectioning compound (Sakura Finetek, Japan), and flash frozen in liquid N_2_. Tissue blocks were cut into 10 µm-thick sections. The other half liver lobe were excised and flash frozen in liquid N_2_ for RNA extraction.

### Plasma Lipid and Lipoprotein Fractionation

Plasma lipoproteins were fractionated by fast protein liquid chromatography (FPLC) on a Superose 6 GL 10/30 column (GE Healthcare, NJ). For FPLC, plasmas were pooled from 7 mice/group prior to fractionation. Colorimetric assays were used to measure cholesterol in plasma and FPLC fractions according to the manufacturer’s instructions (Cholesterol E, Wako, VA). Colorimetric assays were measured with a VersaMax microplate reader (Molecular Devices Corporation, Sunnyvale, CA).

### Western Blot Analysis of apoE and apoB

Plasma and FPLC fractions (from 4 and 20 week-old mice) were subjected to SDS-polyacrylamide gel electrophoresis (SDS-PAGE; 3–15% gels) and transferred to nitrocellulose membranes (Bio-Rad Laboratories, CA). Plasma samples from 4 (Baseline) and 20 week-old mice (16-week HCD) were also subjected to SDS-PAGE on 12% mini-gels and transferred to nitrocellulose membranes. Western blots were incubated with primary antibodies: rabbit anti-mouse apoE (1∶10,000, [37]) and apoB (1∶10,000, [28]), followed by detection with IRDye 680 LT goat anti-rabbit antibody (1∶5000, LI-COR Biosciences, NE). Membranes were scanned and quantified on the Odyssey infrared imaging system (LI-COR Biosciences, NE). Values were expressed as fold-increased.

### Histological Quantification of Atherosclerosis

Beginning at the base of the aortic root, sections were cut at 10 µm thickness, collected, and arranged in 3 sections per slide. Atherosclerotic lesions in the aortic root were quantified by staining with oil-red O to reveal neutral lipids in 3 cross-sections, 50 µm apart, and counterstained with hematoxylin. Adjacent sections were stained with Sirius red counterstained with Fast-green to reveal collagen. Slides were mounted on a Zeiss AxioObserver Z1 microscope (Carl Zeiss Microimaging inc., Thornwood, NY) and images captured with a Retiga-SRV CCD camera equipped with RGB color filter (Qimaging, Surrey, BC, Canada). Collagen types I and III were visualized with a circular polarizer. Surface areas were quantified with Metamorph software (Molecular Devices Inc).

### Immunofluorescence Characterization of Aortic Root Lesions and Liver Sections

Formalin fixed and glycine quenched cross-sections of the aortic root and liver were permeabilized for 5 min with 0.5% Saponin in PBS. Non-specific labeling was blocked with 10% donkey serum in PBS with 0.1% Saponin for 2 h at RT. Aortic root sections were simultaneously incubated with rat anti-Mac-2 (1∶1000, Cedarlane Labs Ltd., ON, CA) and rabbit anti-apoE (1∶200) overnight at 4°C or mouse anti-smooth muscle α-actin antibody conjugated to Cy3 (1∶200) for 2 hrs at RT in antibody buffer solution (solution of sodium citrate (SSC) supplemented with 2% donkey serum, 0.1% BSA and 0.1% Saponin). Liver sections were simultaneously incubated with rabbit anti-apoE (1∶1000) and either rat anti-Mac-2 (1∶1000) or goat anti-VE-Cadherin (1∶200, Santa Cruz Biotechnology, Inc., CA). After three 5 min-washes with 0.1% Saponin SSC, sections were incubated in antibody buffer containing Alexa488 conjugated donkey anti-rat or anti-goat and Alexa594 conjugated anti-rabbit secondary antibodies (1∶1000; Invitrogen) for 2 h at RT. Following a 3×5 min-wash with 0.1% Saponin SSC, the nuclei were stained with Hoechst 33342 (1∶5,000; Invitrogen) and slides mounted in SlowFade Gold (Invitrogen). Images of each combination of up to triple labels were acquired with a Zeiss AxioObserver microscope (as described above) and images quantified with Metamorph software (Molecular Devices Inc). For individual cell immunofluorescence intensity quantification, a region of interest was first drawn using as a guide, a cell specific marker (Mac-2 for Kupffer cells and non-Mac-2 for hepatocytes) and the mean fluorescence intensity for this region calculated. For each mouse, we averaged the results from 10 randomly chosen cells. Controls for cross-reactivity between primary antibody and secondary antibody of a different species were all negative. Clear separation of each channel was also verified by omitting one of the primary antibodies.

### In-vitro Peritoneal Macrophage Cell-culture

Peritoneal macrophages were extracted from C57BL/6J, *Apoe^h/h^ LysM-Cre* and *Apoe^h/h^* mice after treatment with Concanavalin A and plated in 6-well tissue culture plates. Cells were allowed to grow to confluence in Dulbecco’s Modified Eagle Medium (DMEM; Cellgro, Manassas, VA) supplemented with 20% L-Cell Conditioned Media, 10% fetal bovine serum (FBS, Invitrogen). The cells were first incubated for 48 h with 50 µg/mL of human acetylated LDL (acLDL) (BTI, Stoughton, MA) and for 24 h with 3 µM of LXR agonist T0901317 (Cayman Chemical, Ann Arbor, MI) then incubated in DMEM supplemented with 10% Lipoprotein depleted bovine serum for 60hrs, in the absence of exogenous apoE. Media’s were collected and cells lysed with RIPA buffer.

### RNA Extraction and Isolation

RNA was extracted from flash frozen livers and cultured peritoneal macrophages using the RNeasy Mini Kit (Qiagen Inc., CA), with a DNase step according to the manufacturer’s instructions. Liver tissue homogenization was performed with a Tissue-Tearor (Biospec Products Inc, OK). Quantities of RNA were measured using NanoDropTM 2000 (Thermo Scientific, DE).

### Analysis of Gene Expression by Quantitative Real-time RT-PCR

Quantitative Real-time reverse transcriptase polymerase chain reaction (qRT-PCR) was used to analyze gene expression levels in cultured peritoneal macrophages. Gene expression was determined with an ABI Prism 7900 (Applied Biosystems, Foster City, CA) using in house designed primer pairs and SyBr green reactions (Applied Biosystems). cDNA was reverse-transcribed from 100ng total RNA (iScript, Bio-Rad Laboratories). Two µL of diluted (1∶10) cDNA template were used in each qRT-PCR reaction. ApoE gene expression was normalized to the housekeeping gene Cyclophilin A and calculated according to the 2^−ΔΔCt^ method. All experiments were performed in triplicate.

### Statistical Analysis

Data were analyzed with GraphPad Prism 5 software (GraphPad Software Inc., La Jolla, CA) using two-tailed Student *t* tests unless otherwise stated. A difference with a *P value of* <0.05 was considered significant.
